# The Expenditure at the Mary Wardell Convalescent Home

**Published:** 1897-09-04

**Authors:** 


### EDITOR'S LETTER-BOX.

[Our Correspondents are reminded that prolixity is a great bar to publication, and that brevity of style and conciseness of statement greatly facilitate early insertion.]

### THE EXPENDITURE AT THE MARY WARDELL CONVALESCENT HOME.

Miss Wardell writes: I observe in your issue of August 14th a criticism of the expenditure of this home, "the cost per patient" being "relatively high—we think too high"—£2 10s. to £3 10s. per week. I think the fact that this home differs greatly from any other ordinary convalescent home is not kept in mind either by your critic or by the Hospital Sunday Fund Committee in their distribution of grants. An ordinary convalescent home provides nothing more than a house, with a bed and wholesome food for the patients who resort to it for rest and change of air. The case of my home is far different. (1) A man, horse, and omnibus have to be provided, and besides the cost of their maintenance at the home, each time the omnibus is sent to town for a patient the man has to put up at some place for rest, and bait has to be paid for. (2) As the patient cannot go about the neighbourhood on account of risk of spread of infection, grounds for their enjoyment of outdoor exercise have to be provided and kept in good order. This involves the employment of two gardeners. (3) As the patients cannot send their soiled linen to their friends or to a laundress a laundry has to be maintained by the home, involving the expense of two laundry maids, with fuel, soap, and all laundry appliances. (4) As the patients cannot be discharged without a medical certificate to the effect they are safe to mix with the general public, and as scarlet fever is followed during convalescence by many serious results, if not carefully watched, a visiting medical officer has to be employed, at a salary, and a trained nurse, also at a good salary, has to be maintained in the home, besides a costly supply of drugs and disinfectants, surgical appliances for ears, noses, &c. (5) The home being necessarily in an isolated position, an expensive system of drainage had to be laid out, and to be maintained in full efficiency. The distance from town or village also renders the lighting of the home more expensive, and a man has to be employed to trim, clean, and fill lamps for the whole building and outbuildings, to attend to the heating apparatus in winter, to the furnace for hot-water supply, and steam disinfecting apparatus, &c. All these very heavy expenses have to be reckoned before we come to the cost of actual maintenance of the patients, which is the only charge on an ordinary convalescent home. The adult patients and the doctors who occasionally call at the home are unanimous in remarking that the cost of each patient must exceed the sum of £3 3s. a week, and this cost would be still higher if there were secretary's salary and other office expenses to meet. Up to the present time my voluntary labour has spared the home this additional burden. I shall be grateful if you will give publicity to this statement of facts, which I hope may reach those who at present seem ignorant of the great difficulties with which such a special home as this has to contend, and who form their judgment of its expenditure without having looked into its work. I could point out other reasons for "high costs," such as the fluctuating numbers, &c., but my letter is already too long.

